# Factors associated with caregiver burden of toileting assistance at home versus in a nursing home: A cross-sectional study

**DOI:** 10.1371/journal.pone.0299721

**Published:** 2024-03-07

**Authors:** Miho Shogenji, Mikako Yoshida, Takahiro Kakuchi, Kohei Hirako

**Affiliations:** 1 Faculty of Health Sciences, Institute of Medical, Pharmaceutical and Health Sciences, Kanazawa University, Kanazawa City, Ishikawa, Japan; 2 Department of Women’s Health Nursing & Midwifery, Tohoku University Graduate School of Medicine, Sendai City, Miyagi, Japan; 3 Graceful Aging Division, Health & Welfare Department, Komatsu City Hall, Komatsu City, Ishikawa, Japan; 4 The Establishment Preparation Office for The Faculty of Interdisciplinary Economics, Kinjo University, Hakusan City, Ishikawa, Japan; Cranfield University, UNITED KINGDOM

## Abstract

This study aimed to identify differences in caregiver burden related to toileting assistance, and examine the factors associated with the most burdensome aspects of providing toileting assistance. In 2019, a self-administered postal survey was conducted with 743 caregivers of older adults who received subsidies for continence products in Komatsu City, Japan. Both family caregivers and nursing home staff answered questions regarding older adults’ urinary/fecal symptoms, toileting assistance, and perceived caregiver burden. Older adults living at home had less need for toileting assistance than those in nursing homes. However, family caregivers experienced more burden than nursing home staff. The most frequent physical burden associated with toileting assistance for family caregivers was urinary/fecal leakage from absorbent incontinence products. This burden was linked to family caregivers providing care at home, using a combination of urinary pads and diapers, and symptoms that caused burdens on caregivers including urinary/fecal incontinence, nocturia, and no desire to urinate. These results suggest that leakage caused by the inappropriate use of urinary pads combined with diapers is a source of caregiver burden. Continence care experts should provide guidance to family caregivers of older adults, particularly those who are underweight and frail, regarding the selection and fitting of absorbent incontinence products.

## Introduction

Incontinence includes urinary incontinence (UI), fecal incontinence (FI), and double incontinence (UI and FI) [1, 2]. The occurrence of “no desire to urinate/defecate” is likely with aging, declining physical/cognitive function, dementia, and aphasia. Older adults may become unable to communicate their need to urinate/defecate to others or recognize it for themselves, resulting in frequent incidents of incontinence. The reported prevalence of older adults requiring care owing to incontinence varies widely between studies, with UI, FI, and double incontinence ranging from 10%–77%, 1%–57%, and 4%–65.4%, respectively [3–10].

Caregivers of older adults with incontinence bear the burden of providing toileting assistance. Zarit et al. [11] defined caregiver burden as the extent to which caregivers experience harm to their physical health and emotional and financial well-being as a result of caring for older adults. Toileting assistance includes assistance with tasks such as going to the toilet, sitting on the toilet seat, changing absorbent incontinence products, and cleaning the genital area. These tasks need to be performed several times a day, including at night. If urine or stool leaks from absorbent incontinence products, additional help may be needed, such as washing clothes and managing laundry.

Caregivers of older adults with incontinence strive to provide sensitive assistance to maintain their self-esteem, despite the odor of urine/feces. However, cognitive decline may cause older adults to be restless during urination/defecation or refuse assistance, which places an emotional burden on caregivers. Supporting older adults with incontinence also places a burden on caregivers by preventing them from leaving the home or working outside the home [12–17]. In addition, caregivers bear the financial burden of purchasing absorbent incontinence products on a daily basis.

Moreover, toileting assistance is associated with increased caregiving time [12], decreased sleep time [13], increased perceived caregiver burden [14–16], and decreased quality of life (QOL) [17]. Therefore, it is important to identify ways to manage caregiver burden. However, the specific type of toileting assistance that is the most burdensome and the factors associated with it remain unclear.

The severity and content of activities of daily living (ADLs) related to toileting that result in caregiver burden vary between the home and nursing home settings. For example, older adults with severe incontinence and those who require extensive assistance may have difficulty managing with family caregivers at home and may be admitted to a nursing home [9]. Thus, caregiver burden for toileting assistance may differ between family caregivers who provide the frequent care that is required throughout the day at home and nursing home staff who provide care during their working hours.

Although caregiver burden increases and QOL decreases due to providing toileting assistance, healthcare providers generally tend to focus on older adult patients. Therefore, we felt it was necessary to evaluate the specific type of toileting assistance that causes burdens on caregivers. Thus, the aim of this study was to clarify the differences in caregiver burden of toileting assistance between family caregivers at home and nursing home staff and examine factors associated with the toileting status and assistance that is the most burdensome for caregivers.

## Materials and methods

### Study design and participants

A self-administered postal questionnaire survey was conducted in Komatsu City, Japan, from November to December 2019. Study participants were caregivers of older adults with incontinence who received government subsidies for continence products in Komatsu City, Japan, in 2019. This service is widespread in Japan, with most local governments providing continence products or financial support to older adults who have UI or FI and require assistance with most ADLs because of physical or cognitive decline caused by disease, disability, or aging.

Questionnaires were mailed to the residential addresses of the older adults. Primary family caregivers completed the questionnaire if the older adults lived at home. For older adults living in nursing homes, family members asked the nursing home staff who provided toileting assistance to complete the questionnaire.

This study was approved by the Kanazawa University Medical Ethics Review Committee (No. 924–1). A research disclosure form was published on the Komatsu City Hall website to provide information about the study. Informed consent was obtained from family members of older adults, who confirmed their consent after the study was explained to them.

### Variables

#### Characteristics of older adults

Data on age, sex, duration of long-term care, physical/cognitive function, and level of toileting assistance were extracted from the long-term care insurance claims data of Komatsu City employees. Physical function was classified into three levels: independent, house-bound, and chair-bound/bed-bound, according to the “Criteria for determination of the daily life independence level (bedridden level) of the elderly with disability.” Cognitive function was classified into two levels (independent or declined) using the “Criteria for determination of the daily life independence level of the elderly with dementia” [18]. The degree of toileting assistance was classified into four levels: independent, requires partial assistance (verbal instruction or physical assistance by a caregiver), requires almost complete assistance (physical assistance by one or two caregivers), and requires constant assistance.

#### Toileting status and caregiver burden

We asked about older adults’ toileting status and the burden on caregivers arising from providing toileting assistance in the last month. The toileting status of older adults included the location of toileting (toilet or bedside commode, on the bed), continence products (absorbent incontinence products, bedside commode, urinal, and indwelling catheter), and toileting behaviors requiring assistance. If absorbent incontinence products were used, the type (tape-type diaper, pant-type diaper, urinary pad) and use of absorbent incontinence products (urinary pad combined with diaper, diaper/urinary pad only, or none) were assessed. Caregivers were asked to indicate whether they found the following symptoms by older adults to be burdensome: urinary symptoms (UI, frequent urination, voiding symptoms, and no desire to urinate) and fecal symptoms (FI, diarrhea, constipation, and no desire to defecate). Caregivers were asked to indicate whether they found the following aspects of toileting assistance to be burdensome: assistance with movement, nighttime/frequent assistance, urinary/fecal leakage from absorbent incontinence products, guidance to location/procedure of toileting, the odor of urine/feces, refusal of assistance, cost/availability of absorbent incontinence products, and disposal of used absorbent incontinence products. These items were developed by a nurse researcher, who is an expert in continence care, through group discussions with local hospitals and home healthcare continence care experts.

### Analysis

The researcher matched and linked the questionnaire data with long-term care insurance claims data using an anonymized study ID. Differences in burdensome toileting assistance between family caregivers and nursing home staff were examined using the Mann–Whitney U test, Pearson’s chi-square test, and Fisher’s exact test.

Binomial logistic regression analysis with forced entry of variables (p < 0.05) in the univariate analysis was used to examine the factors associated with the most burdensome toileting assistance. Variables were selected by reviewing the multicollinearity of the independent variables before imputation.

JMP ver. 16.2 (SAS Japan) was used for statistical analysis. The significance level was set at p < 0.05 for two-sided tests.

## Results

### Summary of participants

**Fig 1:**
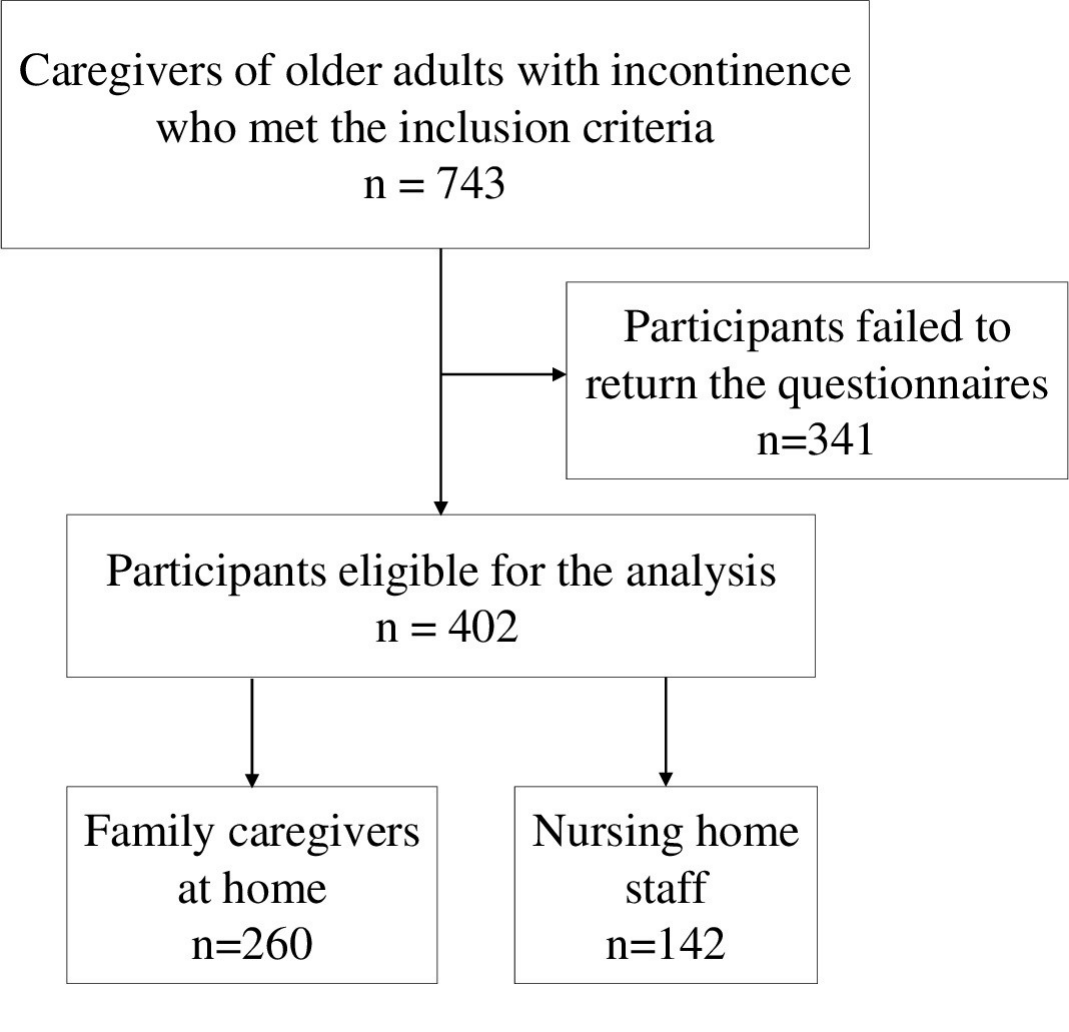
Flowchart of participant selection criteria.

Of the 743 caregivers, 402 responded to the questionnaire; 260 and 142 were family caregivers at home and nursing home staff, respectively (Fig 1).

Compared with older adults in nursing homes, older adults at home had a lower median age (85 years vs. 90 years, p < 0.001) and median duration of long-term care (6 years vs. 7 years, p = 0.012). The proportion of females (54.2% vs. 76.8%, p < 0.001) and older adults with cognitive decline (83.5% vs. 93%, p = 0.007) were lower among older adults at home than among those in nursing homes.

### Differences in toileting status and caregiver burden of toileting assistance between home and nursing homes

Ninety-nine percent of older adults receiving care from the participants in our survey used absorbent incontinence products. Compared with older adults in nursing homes, the proportions of older adults at home using pant-type diapers (78.5% vs. 62.7%, p < 0.001) and bedside commodes were higher (25.4% vs. 7.8%, p < 0.001).

Regarding toileting assistance, the home group had lower proportions of individuals who required constant assistance (53.5% vs. 68.3%, p = 0.008). They experienced fewer problems with toileting procedures including no desire to urinate/defecate (22.7% vs. 33.8%, p = 0.016), unable to understand the location/procedure of toileting (22.7% vs. 40.1%, p < 0.001), and unable to wear underclothes and pants independently (49.2% vs. 60.6%, p = 0.030) (Table 1).

**Table 1:** Demographics and toileting status of older adults at home or in nursing homes in the univariate analysis.

N = 402		Home		Nursing home		p-value
		n = 260		n = 142		
Age (years), median (IQR)		85	(78.3–91)	90	(84.8–92)	< 0.001
Female, n (%)		141	(54.2)	109	(76.8)	< 0.001
Long-term care duration (years), median (IQR)		6	(4–10)	7	(5–11)	0.012
Physical function						
	Independent, n (%)	82	(31.5)	47	(33.1)	0.772
	House-bound, n (%)	123	(47.3)	62	(43.7)	
	Chair-bound or bed-bound, n (%)	55	(21.2)	33	(23.2)	
Cognitive function						
	Independent, n (%)	43	(16.5)	10	(7.0)	0.007
	Declined, n (%)	217	(83.5)	132	(93.0)	
Degree of toileting assistance						
	Independent, n (%)	3	(1.2)	4	(2.8)	0.008
	Requires partial assistance, n (%)	5	(1.9)	3	(2.1)	
	Requires almost complete assistance, n (%)	113	(43.5)	38	(26.8)	
	Requires constant assistance, n (%)	139	(53.5)	97	(68.3)	
Location of toileting						
Toilet or bedside commode, n (%)		178	(68.5)	88	(62.0)	0.189
On the bed, n (%)		82	(31.5)	54	(38.0)	
Use of continence products						
	Tape-type diaper, n (%)	113	(43.5)	80	(56.3)	0.014
	Pant-type diaper, n (%)	204	(78.5)	89	(62.7)	< 0.001
	Urinary pad, n (%)	227	(87.3)	133	(93.7)	0.047
	Bedside commode, n (%)	66	(25.4)	11	(7.8)	< 0.001
	Urinal, n (%)	18	(6.9)	1	(0.7)	0.005
	Indwelling catheter, n (%)	13	(5.0)	3	(2.1)	0.157
Toileting behaviors that need assistance						
	No desire to urinate/defecate, n (%)	59	(22.7)	48	(33.8)	0.016
	Unable to understand the location/procedure of toileting, n (%)	59	(22.7)	57	(40.1)	< 0.001
	Unable to move between bedroom and bathroom, n (%)	124	(47.7)	76	(53.5)	0.264
	Unable to wear underclothes and pants independently, n (%)	128	(49.2)	86	(60.6)	0.030
	Unable to sit in the toilet seat, n (%)	95	(36.5)	65	(45.8)	0.071
	Unable to urinate or defecate, n (%)	62	(23.9)	40	(28.2)	0.341
	Unable to clean the genital area, n (%)	133	(51.2)	84	(59.2)	0.124

Mann–Whitney U test, Pearson’s chi-square test, Fisher’s exact test

**Fig 2:**
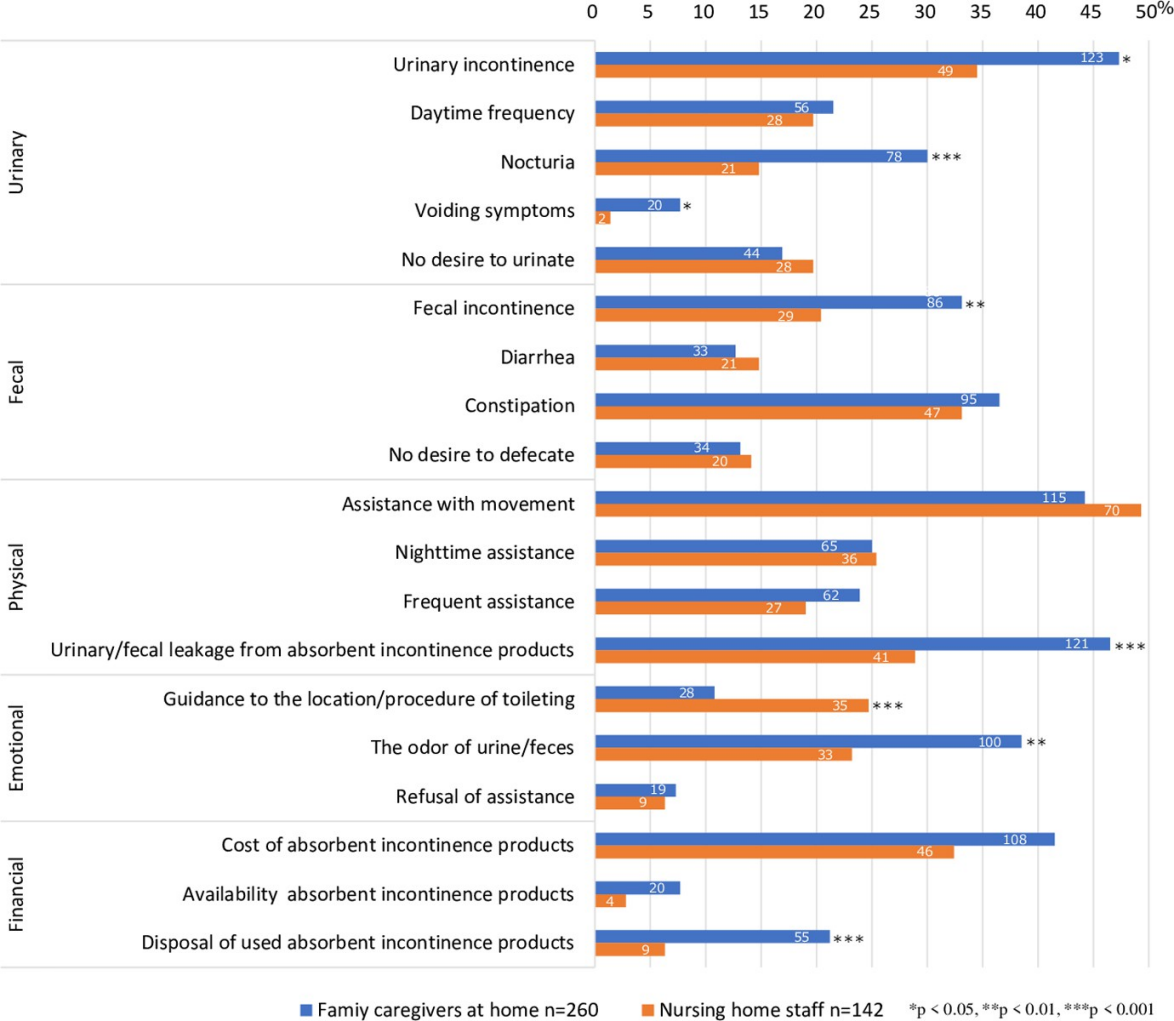
Differences in the burden of toileting assistance between family caregivers at home and nursing home staff.

Compared to nursing home staff, family caregivers perceived a statistically greater burden associated with the older adults’ symptoms of UI, nocturia, voiding symptoms, and FI; assisting with toileting when urine or stool leaks from absorbent incontinence products; dealing with the odor of urine/feces; and disposing of used absorbent incontinence products (p < 0.05, Fig 2).

### Factors associated with urinary/fecal leakage from absorbent incontinence products

Urinary/fecal leakage from absorbent incontinence products was the most frequent physical burden associated with toileting assistance for family caregivers (46.5%). Therefore, we examined the factors associated with this problem (Fig 2).

Of the 162 caregivers who experienced the burden of leakage from absorbent incontinence products, 100 (61.7%) experienced the burden of UI, 70 (43.2%) experienced the burden of FI, and 20 (12.3%) experienced the burden of both FI and diarrhea. Compared to those without this burden, family caregivers who experienced burden by leakage from absorbent incontinence products were more likely to care for older adults who used urinary pads combined with diapers and had symptoms of nocturia, no desire to urinate, and diarrhea. Both family caregivers and nursing home staff with this burden tended to express UI/FI and the odor of urine/feces as sources of burden (Table 2).

**Table 2:** Toileting status and assistance associated with urinary/fecal leakage from absorbent incontinence products on univariate analysis.

			Family caregivers n = 260					Nursing home staff n = 142				
N = 402			Burdened by leakage from absorbent incontinence products n = 121		Not burdened by leakage from absorbent incontinence products n = 139		p-value	Burdened by leakage from absorbent incontinence products n = 41		Not burdened by leakage from absorbent incontinence products n = 101		p-value
Age (years), median (IQR)			86	(79–91)	84	(78–91)	0.178	89	(81.5–93)	90	(85.5–92)	0.961
Women, n (%)			62	(51.2)	79	(56.8)	0.366	31	(75.6)	78	(77.2)	0.836
Use of continence products												
		Tape-type diaper, n (%)	51	(42.2)	62	(44.6)	0.690	21	(51.2)	59	(58.4)	0.433
		Pant-type diaper, n (%)	94	(77.7)	110	(79.1)	0.777	29	(70.7)	60	(59.4)	0.206
		Urinary pad, n (%)	116	(95.9)	111	(79.9)	< 0.001	41	(100)	92	(91.1)	-
		Bedside commode, n (%)	27	(22.3)	39	(28.1)	0.289	1	(2.4)	10	(9.9)	0.132
Combination of absorbent incontinence products												
		Urinary pad combined with diaper, n (%)	114	(94.2)	110	(79.1)	< 0.001	39	(95.1)	90	(89.1)	0.348
Urinary/fecal symptoms and toileting assistance as sources of burden												
	Urinary	Urinary incontinence, n (%)	74	(61.2)	49	(35.3)	< 0.001	26	(63.4)	23	(22.8)	< 0.001
		Daytime frequency, n (%)	28	(23.1)	28	(20.1)	0.558	8	(19.5)	20	(19.8)	0.969
		Nocturia, n (%)	45	(37.2)	33	(23.7)	0.018	7	(17.1)	14	(13.9)	0.625
		Voiding symptoms, n (%)	11	(9.1)	9	(6.5)	0.430	0	(0)	2	(2.0)	-
		No desire to urinate, n (%)	31	(25.6)	13	(9.4)	< 0.001	12	(29.3)	16	(15.8)	0.068
	Fecal	Fecal incontinence, n (%)	55	(45.6)	31	(22.3)	< 0.001	15	(36.6)	14	(13.9)	0.002
		Diarrhea, n (%)	21	(17.4)	12	(8.6)	0.035	8	(19.5)	13	(12.9)	0.312
		Constipation, n (%)	47	(38.8)	48	(34.5)	0.472	17	(41.5)	30	(29.7)	0.177
		No desire to defecate, n (%)	20	(16.5)	14	(10.1)	0.124	9	(22.0)	11	(10.9)	0.086
	Physical	Assistance with movement, n (%)	56	(46.3)	59	(42.5)	0.535	20	(48.8)	50	(49.5)	0.938
		Nighttime assistance, n (%)	34	(28.1)	31	(22.3)	0.282	15	(36.6)	21	(20.8)	0.050
		Frequent assistance, n (%)	34	(28.1)	28	(20.1)	0.133	5	(12.2)	22	(21.8)	0.241
	Emotional	Guidance to the location/procedure of toileting, n (%)	14	(11.6)	14	(10.1)	0.698	13	(31.7)	22	(21.8)	0.214
		The odor of urine/feces, n (%)	62	(51.2)	38	(27.3)	< 0.001	15	(36.6)	18	(17.8)	0.016
		Refusal of assistance, n (%)	8	(6.6)	11	(7.9)	0.687	3	(7.3)	6	(5.9)	0.718
	Financial	Cost of absorbent incontinence products, n (%)	57	(47.1)	51	(36.7)	0.089	19	(46.3)	27	(26.7)	0.024
		Availability of absorbent incontinence products, n (%)	9	(7.4)	11	(7.9)	0.886	2	(4.9)	2	(2.0)	0.579
		Disposal of used absorbent incontinence products, n (%)	29	(24.0)	26	(18.7)	0.300	5	(12.2)	4	(4.0)	0.121

Mann–Whitney U test, Pearson’s chi-square test, Fisher’s exact test

Urinary/fecal leakage from absorbent incontinence products was associated with family caregivers providing care at home (adjusted odds ratio [AOR]:1.896, 95% confidence interval [CI]:1.147–3.133, p = 0.013); use of urinary pads combined with diapers (AOR:2.837, 95%CI:1.262–6.377, p = 0.012); UI (AOR:2.604, 95%CI:1.628–4.163, p<0.001); nocturia (AOR:1.802, 95%CI:1.076–3.017, p = 0.025); no desire to urinate (AOR:2.187, 95%CI:1.221–3.919, p = 0.009); and FI (AOR:1.822, 95%CI:1.083–3.065, p = 0.024), even after adjustment for other factors (Table 3).

**Table 3:** Factors associated with experiencing burden from managing urinary/fecal leakage from absorbent incontinence products at home and in nursing homes.

N = 402	AOR	(95%CI)			p-value
Age	1.487	(0.684	—	3.233)	0.316
Sex	0.758	(0.471	—	1.221)	0.255
Caregivers	1.896	(1.147	—	3.133)	0.013
Urinary pad combined with diaper	2.837	(1.262	—	6.377)	0.012
Urinary/fecal symptoms as sources of burden					
Urinary incontinence	2.604	(1.628	—	4.163)	<0.001
Nocturia	1.802	(1.076	—	3.017)	0.025
No desire to urinate	2.187	(1.221	—	3.919)	0.009
Fecal incontinence	1.822	(1.083	—	3.065)	0.024
Diarrhea	1.381	(0.713	—	2.674)	0.338

Age (Under 75 years: 0, Over 75 years: 1); Sex (Male: 0, Female: 1); Caregivers (Nursing home staff: 0, Family caregivers: 1); Urinary pad combined with diaper (No: 0, Yes: 1); Urinary incontinence (No: 0, Yes: 1); Nocturia (No: 0, Yes: 1); No desire to urinate (No: 0, Yes: 1); Fecal incontinence (No: 0, Yes: 1); Diarrhea (No: 0, Yes: 1).

Binomial logistic regression analysis, AOR: Adjusted odds ratio, CI: Confidence interval

Tests for the whole model: P <0.001. Contribution rate: 0.150. Lack of fit: p = 0.082.

## Discussion

This study examined differences in the burden of toileting assistance between family caregivers at home and nursing home staff members. Older adults at home were less likely to require toileting assistance than those in nursing homes. However, family caregivers experienced more burden than nursing home staff. The most frequent physical burden associated with toileting assistance for family caregivers was urinary/fecal leakage from absorbent incontinence products. This was associated with family caregivers providing care at home, using urinary pads combined with diapers, and older adults’ symptoms that caused caregivers to experience burdens, including UI/FI, nocturia, and no desire to urinate.

In this study, although older adults living at home had less need for toileting assistance than those in nursing homes, family caregivers experienced a greater burden than the nursing home staff. This may be because family caregivers are responsible for providing frequent toileting care throughout the day, resulting in chronic sleep disruption and exhaustion [19]. Nursing home staff, despite the difficulty of assisting many older adults, may consider it to be part of their job. In comparison, family caregivers may experience a greater sense of responsibility and burden because of a moral obligation to their family. In addition, family caregivers have limited opportunities to consult with experts on incontinence care [13, 20], even though motor/cognitive disabilities in older adults gradually worsen over time. Without appropriate guidance from these experts, family caregivers are more likely to feel burdened.

Consistent with a previous study reporting that incontinence causes caregiver burden [15], the present study found that urinary/fecal leakage from absorbent incontinence products was the most burdensome aspect of providing care. Leakage of urine or stool from absorbent incontinence products adds physical and emotional stress to the caregiver burden, including the challenges of dealing with soiled laundry and unpleasant odors. Moreover, irregular leakage from absorbent incontinence products makes caregivers feel as though they are doing extra work and are unable to prevent the leakage, leading to frustration or irritation.

Diapers are more expensive than urinary pads and require more effort to change on the bed. Attaching a urinary pad to a diaper and changing it after only one or more incontinence cycles is a popular practice in Japan to reduce physical and financial burdens. In this study, the use of urinary pads combined with diapers had the strongest association with urinary/fecal leakage from absorbent incontinence products, suggesting that the use of urinary pads combined with diapers may not reduce physical and financial burdens. The discrepancy between caregivers’ expectations of a reduction in burden with the use of urinary pads combined with diapers and our findings may have occurred for two reasons. First, caregivers may struggle to fit urinary pads and diapers properly around the hip joint without gaps, even if the hip bones become severely prominent. Additionally, many older adults who require nursing care are underweight and frail. They have muscle weakness due to geriatric syndromes or disuse syndromes [21, 22]. Second, caregivers often use multiple layers of urinary pads due to concerns about leakage. However, each pad has a plastic layer; hence, only the top pad will work to receive urine. When multiple pads are placed inside the diaper, the height of the three-dimensional gathers of the diaper is less than that of the pads. This results in diapers that cannot hold urine or stool, leading to urinary/fecal leakage. Therefore, using diapers alone without pads may be a solution when caregivers are burdened with urinary/fecal leakage from absorbent incontinence products.

Both UI and FI are associated with urinary/fecal leakage from absorbent incontinence products. Urinary leakage depends on the pad’s shape and absorption amount and the speed of urine flow. Even a high-absorbent urinary pad may not absorb urine quickly enough if the urine flow is too fast [23]. In some countries, tight-fitting mesh pants are used in many care settings to hold urinary pads in place, but such pants are not commonly used in Japan. Selecting diapers that are the correct size for the older adult wearing them and choosing the appropriate type to manage volume and urine flow rate is important. Furthermore, fecal leakage tends to occur after visiting home nurses/staff provide constipation care through enemas and digital decompaction [24]. The urinary pad does not effectively absorb loose stool, and fecal leakage can cause skin problems in older adults or increased caregiving time [25]. Therefore, caregivers may benefit from preparing appropriate absorbent incontinence products for older adults after constipation care, as the individual is more likely to have FI and diarrhea. As such, family caregivers need guidance from continence care experts to select appropriate absorbent incontinence products based on the extent and timing of UI and FI in older adults.

This study found that nocturia and no desire to urinate were associated with urinary/fecal leakage from absorbent incontinence products. These lower urinary tract symptoms are also associated with comorbidities in older adults. Nocturia is often caused by nocturnal polyuria [26]. No desire to urinate is associated with nervous system disorders and cognitive dysfunction. Family caregivers in home-care settings have difficulty assessing the toileting status of older adults with no desire to urinate. Therefore, older adults with these symptoms should undergo urological and behavioral therapy provided by healthcare professionals.

This study has several limitations. First, this study was conducted in one region, which poses challenges in generalizing the results to individuals in other cities. However, the ADL and cognitive function of the older adults in this study were similar to those who received long-term home care in other urban regions in Japan [27]. Therefore, our results could reflect the situation of toileting assistance and caregiver burden in older adults who use absorbent incontinence products and require extensive toileting assistance in home care settings. Second, we did not obtain data on diagnosing and treating lower urinary tract or defecation disorders. Hence, we could not examine the proportion of older adults receiving treatment for these disorders and their impact on caregiver burden. Third, we did not assess the extent of care provided by primary caregivers. Some family caregivers may use home healthcare or visiting nurse services during the daytime. Nursing home staff may provide toileting assistance to several residents. Thus, the perceived caregiver burden could not be accurately evaluated for the study participants. Therefore, further studies are needed to investigate the relationship between required toileting assistance and caregiver burden.

## Conclusions

Older adults living at home had less need for toileting assistance than those in nursing homes. However, family caregivers perceived a greater burden from toileting assistance than nursing home staff did. Urinary/fecal leakage from absorbent incontinence products was the most frequent physical burden associated with toileting assistance and was linked to the use of urinary pads combined with diapers. These results suggest that leakage caused by the inappropriate use of urinary pads combined with diapers is a source of caregiver burden. Continence care experts in home care settings should provide guidance to family caregivers of older adults, including those who are underweight and frail, in the selection and fitting of absorbent incontinence products.
